# Phenotypic and Genotypic Characterization of *Klebsiella pneumoniae* Isolated From Retail Foods in China

**DOI:** 10.3389/fmicb.2018.00289

**Published:** 2018-03-01

**Authors:** Shuhong Zhang, Guangzhu Yang, Qinghua Ye, Qingping Wu, Jumei Zhang, Yuanbin Huang

**Affiliations:** ^1^State Key Laboratory of Applied Microbiology Southern China, Guangdong Provincial Key Laboratory of Microbial Culture Collection and Application, Guangdong Open Laboratory of Applied Microbiology, Guangdong Institute of Microbiology, Guangzhou, China; ^2^College of Natural Resources and Environment, South China Agricultural University, Guangzhou, China

**Keywords:** food, *Klebsiella pneumoniae*, biotypes, serotypes, virulence genes, antibiotic resistance, (ERIC-PCR), (GTG)_5_-PCR

## Abstract

*Klebsiella pneumoniae* is not only a major hospital-acquired pathogen but also an important food-borne pathogen that can cause septicaemia, liver abscesses, and diarrhea in humans. The phenotypic and genotypic characteristics of *K. pneumoniae* in retail foods have not been thoroughly investigated in China. The objective of this study was to characterize *K. pneumoniae* isolates through biotyping, serotyping, determination of virulence factors, antibiotic resistance testing, enterobacterial repetitive intergenic consensus-polymerase chain reaction (ERIC-PCR), and (GTG)_5_-PCR molecular typing. From May 2013 to April 2014, a total of 61 *K. pneumoniae* isolates were collected from retail foods in China. Using API 20E test strips, five different biotype profiles were identified among these isolates. The majority of isolates belonged to biochemical profile “5215773” (50 isolates, 80.6%). The capsular serotypes of the 61 *K. pneumoniae* isolates and one reference strain were determined by PCR. Of the seven capsular serotypes tested, four different capsular serotypes were identified. Serotypes K1, K20, K57, and K2 were detected in two, three, two, and one isolates, respectively. Serotypes K3, K5, and K54 were not detected. The presence of 11 virulence genes was assessed by PCR. The most common virulence genes were *fimH* (85.5%), *ureA* (79.0%), *wabG* (77.4%), *uge* (56.5%), and *kfuBC* (29.0%). ERIC-PCR and (GTG)_5_-PCR molecular typing indicated high genetic diversity among *K. pneumoniae* isolates. We identified 60 different ERIC patterns and 56 distinct (GTG)_5_ patterns. Genotypic results indicated that isolates carrying similar virulence factors were generally genetically related. Some isolates from the same geographic area have a closer relationship. The isolates showed high levels of resistance to ampicillin (51/62, 82.2%). Resistance to streptomycin (11/62, 17.7%) and piperacillin (10/62, 16.1%) was also common. The presence of virulent and antibiotic-resistant *K. pneumoniae* in foods poses a potential health hazard for consumers. Our findings highlight the importance of surveillance of *K. pneumoniae* in foods.

## Introduction

*Klebsiella pneumoniae* is an important opportunistic pathogen that causes a variety of infectious diseases in humans, including septicaemia, liver abscesses, diarrhea, and pneumonia (Bi and Xu, 2013; Cao et al., 2014; Guo et al., 2017). It is a well-known hospital-acquired pathogen and associated with increased patient morbidity and mortality (Brisse et al., 2009; Cabral et al., 2012). In addition to the clinical environment, *K. pneumoniae* is frequently found in foods including raw vegetables, powdered infant formula, meat, fish, and street foods, and has been considered as an important food-borne pathogen (Haryani et al., 2007; Sun et al., 2010; Puspanadan et al., 2012; Overdevest et al., 2014; Kim et al., 2015; Davis and Price, 2016). In powdered infant formula, *K. pneumoniae* is included in the hazard identification category “B” according to the FAO and WHO guidelines on microorganisms (FAO-WHO, 2004). In recent years, an increasing number of food-borne outbreaks caused by *K. pneumoniae* have been reported in different countries (Calbo et al., 2011; Tambekar et al., 2011; Zhou et al., 2011; Bi and Xu, 2013; Yu and Zhou, 2013).

*K. pneumoniae* can express a variety of virulence factors including capsules, endotoxins, siderophores, iron-scavenging systems, and adhesins, which have been shown to play important roles in its pathogenesis. Capsule is an important virulence factor, which is involved in at least two pathogenic mechanisms: (1) protection of the bacteria from phagocytosis, and (2) direct inhibition of the host immune response (Kang et al., 2015). Some capsular (K) types, particularly K1, K2, K54, K57, K20, and K5, are often associated with community-acquired invasive pyogenic liver abscess syndrome, septicemia, and pneumonia (Fang et al., 2007; Siri et al., 2011; He, 2012). K1, K2, K20, K54, and K57 are highly virulent in experimental infections in mice and are often associated with severe infections in humans and animals (Turton et al., 2008; Yu et al., 2008; Cheng et al., 2015; Wei et al., 2016). K2 and K5 are frequent causes of metritis in mares and are associated with community-acquired pneumonia (Brisse et al., 2009). K3 is generally associated with rhinoscleroma (He, 2012). Capsule typing is currently the most widely used technique for typing *K. pneumoniae* isolates and exhibits good reproducibility in differentiating clinical isolates (Siu et al., 2011). Several PCRs targeting the *wzy* genes have been developed for capsule typing of *K. pneumoniae* (Turton, 2010; Cheng et al., 2015). Other virulence factors such as the *rmpA* gene (regulator of mucoid phenotype A); *allS* gene (encoding the activator of the allantoin regulon, associated with allantoin metabolism); endotoxin-related genes *wabG, uge*, and *wcaG*; iron acquisition system-related genes *iucB, iroNB, ybtA*, and *kfuBC*; adhesin gene *fimH* (type I fimbriae); and *ureA* gene (α-subunit of the urease, invasin related) are also believed to be involved in virulence processes (Brisse et al., 2009; Turton, 2010; El et al., 2013; Calhau et al., 2014). The detection of such virulence factors is important in understanding the pathogenic characteristics of *K. pneumoniae* isolates and enhancing our knowledge of the health risks posed by this pathogen.

The emergence of antimicrobial resistance in *Klebsiella* spp. isolates is of great concern worldwide in human medicine (Hu et al., 2013). Multidrug-resistant *K. pneumoniae* strains have been isolated from different samples (Nawaz et al., 2012; Falomir et al., 2013; Guo et al., 2016; Yaici et al., 2017). Dietary intake is one of the primary routes for the introduction of antibotic-resistant bacteria and their genes into the human digestive tract. Consumption of specific food categories might influence gut antibiotic resistance gene diversity (Milanović et al., 2017). Moreover, such bacteria may transfer antibiotic resistance determinants to other pathogenic bacteria (Machado et al., 2008). Therefore, surveillance and monitoring of drug-resistant bacteria in foods is important for implementing targeted control strategies and selecting effective drugs for treatment.

Molecular typing is a useful tool for determining the genetic relationships of food-borne bacteria and identifying probable sources of infections. This is particularly important in endemic and epidemic nosocomial outbreaks of *K. pneumoniae* infections to improve the management of such outbreaks. A variety of methods have been used for *K. pneumoniae* typing, including pulsed-field gel electrophoresis (PFGE), enterobacterial repetitive intergenic consensus-polymerase chain reaction (ERIC-PCR), randomly amplified polymorphic DNA (RAPD), and (GTG)_5_ oligonucleotide PCR (Haryani et al., 2007; Ryberg et al., 2011; Barus et al., 2013; Sachse et al., 2014). The ERIC, RAPD, and (GTG)_5_-PCR assays are relatively simple and cost-effective methods that have been successfully used for genotyping *K. pneumoniae* isolates from various sources (Ryberg et al., 2011; Barus et al., 2013).

In recent years, the number of food-borne illness outbreaks caused by *K. pneumoniae* has increased. However, until now, limited information has been available on the characteristics of *K. pneumoniae* isolated from foods. The purpose of the present study was to determine the biotypes, serotypes, virulence genes, and antimicrobial resistance patterns of food isolates and to further analyse their genetic diversity using ERIC-PCR and (GTG)_5_-PCR molecular typing.

## Materials and methods

### Bacterial isolation and biochemical identification

From May 2013 to April 2014, a total of 1,200 retail foods, including 312 ready-to-eat foods (roasted meat, cooked meat, and meatballs), 336 raw meat (poultry, pork, and beef), 192 edible mushrooms (Flammulina velutipes), 240 aquatic products (fish, shrimp, and oysters), and 120 vegetables (cucumber and lettuce), were purchased randomly from supermarkets and farmer's markets in 24 cities of China. The sampling sites covered most of the provincial capitals of China. In each city, 50 samples were randomly collected from two supermarkets and two farmers' markets. The samples were placed in separate sterile plastic bags and then immediately transported to the laboratory in a cooler with ice packs (below 4°C) and processed within 4–6 h.

About 25 g of each food sample was enriched in 225 mL nutrient broth (Huankai Ltd., Guangzhou, China) for 24 h at 37°C. Thereafter, the enrichment was streaked onto MacConkey agar (Huankai Ltd., Guangzhou, China), followed by incubation at 37°C for 24 h. From MacConkey agar, three pink, mucoid colonies were picked up and ubcultured onto nutrient agar at 37°C for 24 h, followed by biochemical identification using API 20 E (BioMe′rieux, Marcy I′Etoile, France). Finally, 61 *K. pneumoniae* isolates were recovered from retail foods. Among these isolates, 12 were from ready-to-eat foods, six were from vegetables, six were from edible mushrooms, 16 were from raw meat, and 21 were from aquatic products. Confirmed cultures were preserved in Luria-Bertani broth containing 20% glycerol and stored at −80°C for further study.

### PCR confirmation of *K. pneumoniae*

All confirmed *K. pneumoniae* isolates were grown overnight in lactose broth at 37°C. Genomic DNA was extracted using a commercial Universal DNA Extraction Kit (Sangon Biotech, Shanghai, China) according to the manufacturer's instructions. Confirmation of *K. pneumoniae* isolates was performed by PCR as previously described (Neuberger et al., 2008). The primer sequences and amplicon size are shown in Table 1. All oligonucleotide primers were synthesized by Sangon Biotech. The PCR mixture (total volume 25 μL) contained 1 × DreamTaqTM Green PCR Master Mix (Fermentas, Waltham, MA, USA), 4 μL primer mixture, and 2 μL DNA template. PCR was conducted in a Bio-Rad PTC-200 Thermal Cycler (Bio-Rad, Hercules, CA, USA). The reference strain *K. pneumoniae* GIM 46117 (khe +) was used as a positive control. The amplified products were analyzed by electrophoresis on 1.5% agarose gels containing Gold View (0.005% v/v) (SBS Genetech, Beijing, China) in 1 × TAE buffer (40 mM Tris–HCl, 1.18 mL acetic acid, 2 mM EDTA, pH 8.0), and the bands were visualized using an ImageQuant 350 Capture system (GE Healthcare, Waukesha, WI, USA).

**Table 1:** PCR primers used in this study.

**Target virulence genes**	**Primer Sequence(5′-3′)**	**Size (bp)**	**References**
*khe*	F: TGATTGCATTCGCCACTGG R: GGTCAACCCAACGATCCTG	428	Neuberger et al., 2008
*wzyK1 (magA)*	F: GGTGCTCTTTACATCATTGC R: GCAATGGCCATTTGCGTTAG	1283	Turton et al., 2008
*wzyK2*	F: GACCCGATATTCATACTTGACAGAG R: CCTGAAGTAAAATCGTAAATAGATGGC	641	Turton et al., 2008
*zxK5*	F: TGGTAGTGATGCTCGCGA R: CCTGAACCCACCCCAATC	280	Turton et al., 2008
*wzyK20*	F: CGGTGCTACAGTGCATCATT R: GTTATACGATGCTCAGTCGC	741	Fang et al., 2007
*wzxK54*	F: CATTAGCTCAGTGGTTGGCT R: GCTTGACAAACACCATAGCAG	881	Fang et al., 2007
*wzyK57*	F: CTCAGGGCTAGAAGTGTCAT R: CACTAACCCAGAAAGTCGAG	1037	Pan et al., 2008
*wzyK3*	F: TAGGCAATTGACTTTAGGTG R: AGTGAATCAGCCTTCACCT	549	Fevre et al., 2011
*rmpA*	F: ACTGGGCTACCTCTGCTTCA R: CTTGCATGAGCCATCTTTCA	535	Brisse et al., 2009
*allS*	F: CCGTTAGGCAATCCAGAC R: TCTGATTTA(A/T)CCCACATT	1090	Brisse et al., 2009
*kfuBC*	F: GAAGTGACGCTGTTTCTGGC R: TTTCGTGTGGCCAGTGACTC	797	Brisse et al., 2009
*ybtA*	F: ATGACGGAGTCACCGCAAAC R: TTACATCACGCGTTTAAAGG	960	He, 2012
*iucB*	F: ATGTCTAAGGCAAACATCGT R: TTACAGACCGACCTCCGTGA	948	He, 2012
*iroNB*	F: GGCTACTGATACTTGACTATTC R: CAGGATACAATAGCCCATAG	992	He, 2012
*fimH*	F: GCTCTGGCCGATAC(C/T)AC(C/G)ACGG R: GC(G/A)(A/T)A(G/A)TAACG(T/C)GCCTGGAACGG	423	Brisse et al., 2009
*ureA*	F: GCTGACTTAAGAGAACGTTATG R: GATCATGGCGCTACCT(C/T)A	337	Brisse et al., 2009
*uge*	F: GATCATCCGGTCTCCCTGTA R: TCTTCACGCCTTCCTTCACT	534	Brisse et al., 2009
*wabG*	F: CGGACTGGCAGATCCATATC R: ACCATCGGCCATTTGATAGA	683	Brisse et al., 2009
*wcaG*	F: GGTTGGKTCAGCAATCGTA R: ACTATTCCGCCAACTTTTGC	169	Turton, 2010

### Serotyping by PCR

The serotypes of 61 *K. pneumoniae* isolates and one reference strain were determined using the PCR-based capsular antigen as previously described (Turton, 2010; He, 2012; Lin et al., 2014; Yu et al., 2015). The primers used are shown in Table 1. These assays differentiate isolates into seven major serovars: K1, K2, K5, K20, K54, K57, and K3.

### Detection of virulence genes of *K. pneumoniae*

Eleven individual PCRs were performed to detect the presence of virulence genes (*rmpA, allS, kfuBC, ybtA, iucB, iroNB, fimH, ureA, uge, wabG*, and *wcaG*) in *K. pneumoniae* isolates as in previous studies (Brisse et al., 2009; Turton, 2010). Primer sequences, amplification conditions, and amplicon sizes are shown in Table 1. Amplified PCR products were analyzed by gel electrophoresis on 1.5% agarose gels containing Gold View (0.005% v/v) in 1 × TAE buffer (40 mM Tris–HCl, 1.18 mL acetic acid, 2 mM EDTA, pH 8.0), and imaged using the ImageQuant 350 Capture system.

### ERIC-PCR

For ERIC-PCR, the primers ERIC1 (5′-ATGTAAGCTCCTGGGGATTCAC-3′) and ERIC2 (5′-AAGTAAGTGACTGGGGTGAGCG-3′) were used (Versalovic et al., 1991). PCR was performed in a 25 μL solution containing 1.0 U of Taq DNA polymerase (Dongsheng Biotech, Guangdong, China), 1.0 μM of each primer, 2.5 mM MgCl_2_, 0.2 mM of each dNTP, and 40 ng of template genomic DNA. Amplifications were performed with a Bio-Rad PTC-200 Thermal Cycler (Bio-Rad) under the following conditions: an initial denaturation at 94°C for 5 min; 35 cycles of 1 min at 94°C, 1 min at 49°C, and 3 min at 72°C; and a final extension at 72°C for 10 min. ERIC-PCR products were detected by a 2.0% agarose gel electrophoresis with Gold View (0.005% v/v) in 1 × TAE buffer (40 mM Tris–HCl, 1.18 mL acetic acid, 2 mM EDTA, pH 8.0), and the gel was photographed using a UV Imaging System (GE Healthcare). The images were captured in TIFF file format for further analysis with BioNumerics software version 6.0 (Applied Maths, Kortrijk, Belgium).

### (GTG)_5_-PCR

(GTG)_5_ oligonucleotide typing was performed as previously described (Ryberg et al., 2011) with minor modifications. The amplification reaction was carried out in a total volume of 25 μL consisting of 1.0 U of Taq DNA polymerase (Dongsheng Biotech,Guangzhou, China), 1.0 μM of primer, 2.5 mM MgCl_2_, 0.25 mM of each dNTP, and 40 ng of template genomic DNA. PCR conditions were as follows: initial denaturation at 94°C for 5 min; 30 cycles of denaturation at 94°C for 30 s, annealing at 49°C for 30 s, and extension at 72°C for 3 min; followed by a final extension at 72°C for 10 min. Genetic relationships among *K. pneumoniae* isolates were analyzed using BioNumerics software version 6.0.

### Antimicrobial susceptibility testing

The strains were tested for antimicrobial susceptibility to 21 antibiotics using the agar disc diffusion method on Mueller–Hinton agar (Oxoid Ltd., Basingstoke, UK) following Clinical and Laboratory Standards Institute (CLSI) guidelines (CLSI (Clinical and Laboratory Standards Institute), 2011). The 21 antibiotics (Oxoid) tested were as follows: ampicillin (10 μg), amoxicillin-clavulanic acid (30 μg), ceftazidime (30 μg), cefotaxime (30 μg), ceftriaxone (30 μg), cefoperazone/sulbactam (105 μg), cephalothin (30 μg), piperacillin (30 μg), piperacillin/tazobactam (110 μg), imipenem (10 μg), cefoxitin (30 μg), kanamycin (30 μg), gentamicin (10 μg), amikacin (10 μg), streptomycin (10 μg), norfloxacin (10 μg), ciprofloxacin (5 μg), nalidixic acid (30 μg), chloramphenicol (30 μg), trimethoprim-sulfamethoxazole (25 μg), and tetracycline (30 μg). These antibiotics are representative of the major classes of antimicrobial drugs important to both veterinary and human medicine. Isolates were classified as susceptible, moderately resistant, and resistant using breakpoints specified by the CLSI, and *Escherichia coli* ATCC 25922 was used as the quality control strain. Moderately resistant isolates were considered resistant.

## Results

### Biochemical and molecular identification of *K. pneumoniae*

A total of five different biochemical profiles were identified among the isolates using API 20E test strips (Table S1). The majority of isolates belonged to biochemical profile “5215773” (97.6% *T* = 1.0; 50 isolates). Other biochemical profiles included “1215773” (LDC-, 98.0%, *T* = 0.93; six isolates), “7215773” (ADH+, 97.5%, *T* = 0.5; three isolates), “5215373” (SOR-, 93.0% *T* = 0.72; two isolates), and “5214773” (VP-, 95.0% *T* = 0.85; one isolate). All isolates were identified as *K. pneumoniae* by PCR.

### Serotypes of *K. pneumoniae*

Capsular serotyping results of the 61 *K. pneumoniae* isolates and one reference strain showed that eight isolates were typeable serotypes and 53 were untypeable serotypes. A total of four different capsular serotypes were identified, with serotype K1 detected in two isolates from fish samples, K20 detected in three isolates (two from chicken and one from mushroom), K57 detected in two isolates (one from shrimp and one from pork), and K2 detected in the reference strain. The other serotypes (K3, K5, and K54) were not detected.

### Distribution of virulence genes

**Figure 1:**
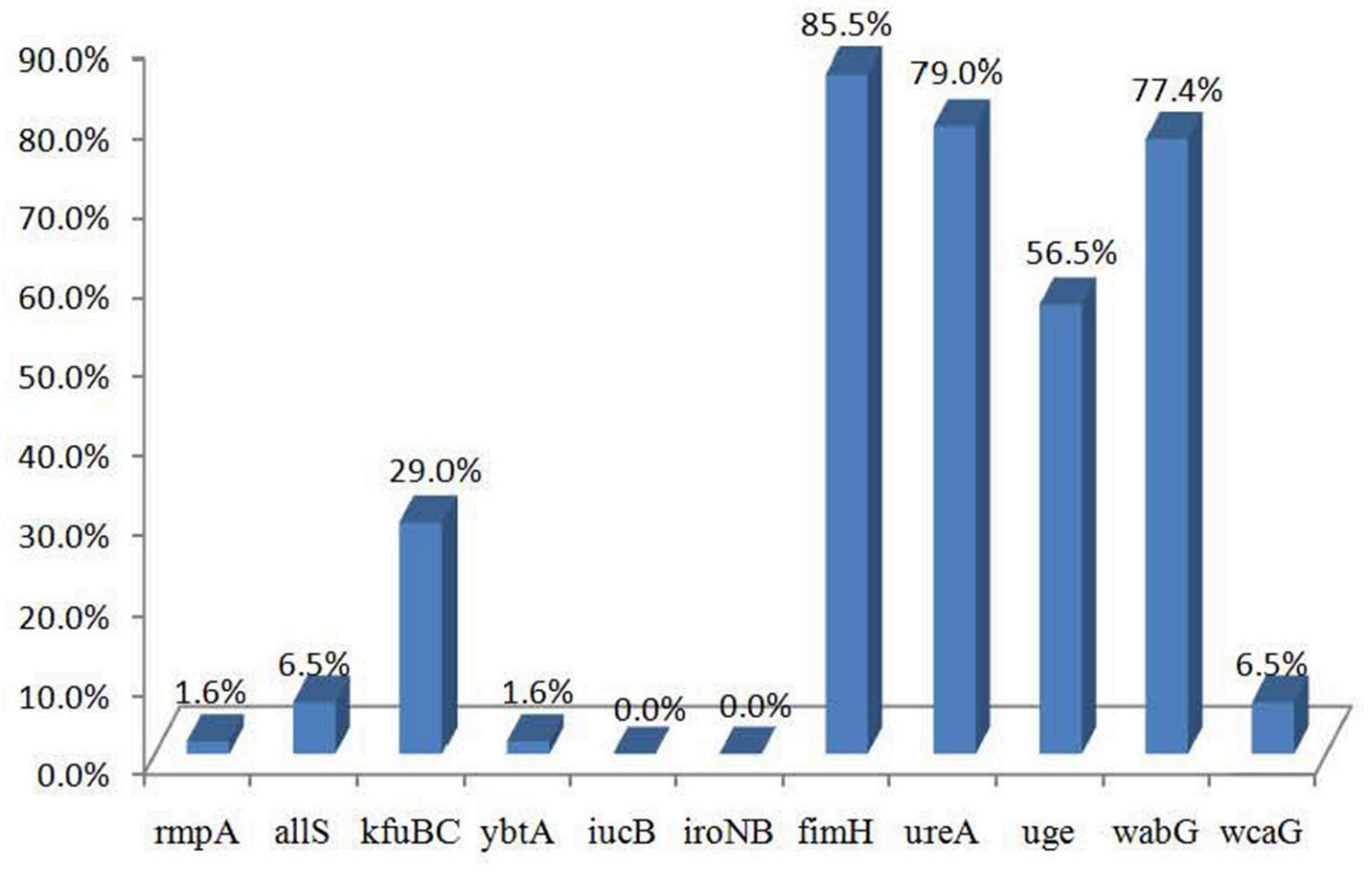
The distribution of virulence genes in *Klebsiella pneumoniae* strains.

The distributions of the 11 virulence genes are shown in Figure 1. Of the 62 strains, 56 carried at least one virulence gene. The virulence genes *fimH, ureA, wabG*, and *uge* were commonly presented in the strains from different sources and detected in 85.5% (53/62), 79.0% (49/62), 77.4% (48/62), and 56.5% (35/62) of the 62 strains, respectively. Virulence genes *kfuBC, allS, wcaG, rmpA*, and *ybtA* were detected in 29.0% (18/62), 6.5% (4/62), 6.5% (4/62), 1.6% (1/62), and 1.6% (1/62) of the strains, respectively, while *iucB* and *iroNB* were not detected.

### Antimicrobial susceptibility testing

The results of antimicrobial susceptibility testing of the 62 strains are shown in Table 2. A high prevalence of resistance to ampicillin (51/62, 82.2%) was observed. Resistance to streptomycin (11/62, 17.7%), piperacillin (10/62, 16.1%), and tetracycline (8/62, 12.9%) was also common. Most of the isolates were susceptible to quinolone and fluoroquinolone antimicrobials including ciprofloxacin, nalidixic acid, and norfloxacin. Some isolates were resistant to β-lactamase antibiotics such as cefotaxime (3/62, 4.8%), cefoxitin (2/62, 3.2%), and amoxycillin-clavulanic acid (2/62, 3.2%). All strains were susceptible to piperacillin/tazobactam, ceftazidime, and imipenem.

**Table 2:** Antibiotic susceptibility of *Klebsiella pneumoniae* strains.

**Antibiotics**	**Antibiotic disc content (μg)**	**Susceptibility**		
		**Resistant No. (%)**	**Intermediate No. (%)**	**Susceptible No. (%)**
**β-LACTAMS**				
Ampicillin (AMP)	10	51 (82.3)	0	11 (17.7)
Amoxycillin-clavulanicAcid (AMC)	30	2 (3.2)	0	60 (96.8)
Ceftazidime (CAZ)	30	0	0	62 (100)
Cefotaxime (CTX)	30	2 (3.2)	1 (1.6)	59 (95.2)
Ceftriaxone(CRO)	30	1 (1.6)	0	61 (98.4)
Cefoperazone/sulbactam(SCF)	105	0	1 (1.6)	61 (98.4)
Cephalothin(KF)	30	7 (11.3)	1 (1.6)	54 (87.1)
Piperacillin(PRL)	100	2 (3.2)	8 (12.9)	52 (83.9)
Piperacillin/tazobactam(TZP)	110	0	0	62 (100)
Imipenem(IPM)	10	0	0	62 (100)
Cefoxitin (FOX)	30	2 (3.2)	0	60 (96.8)
**QUINOLONES AND FLUOROQUINOLONES**				
Ciprofloxacin(CIP)	5	3 (4.8)	1 (1.6)	58 (93.6)
Nalidixic acid (NA)	30	4 (6.5)	2 (3.2)	56 (90.3)
Norfloxacin (NOR)	10	2 (3.2)	1 (1.6)	59 (95.2)
**AMINOGLYCOSIDES**				
Amikacin (AK)	30	0	3 (4.8)	59 (95.2)
Gentamicin (CN)	10	4 (6.5)	1 (1.6)	57 (91.9)
Kanamycin (K)	30	2 (3.2)	2 (3.2)	58 (93.6)
Streptomycin (S)	10	7 (11.3)	4 (6.5)	51 (82.2)
Phenicols				
Chloramphenicol (C)	30	4 (6.5)	0	58 (93.5)
**SULFONAMIDES AND SYNERGISTIC AGENTS**				
Trimethoprim-sulfamethoxazole (SXT)	25	5 (8.1)	0	57 (91.9)
Tetracyclines				
Tetracycline (TE)	30	7 (11.3)	1 (1.6)	54 (87.1)

With regards to multidrug resistance, 17.7% (11/62) of isolates were resistant to three or more of the tested agents. Some of these multidrug-resistant isolates were resistant to 10–15 of these antibiotics (Table 2).

### ERIC-PCR and (GTG)_5_-PCR molecular typing

**Figure 2:**
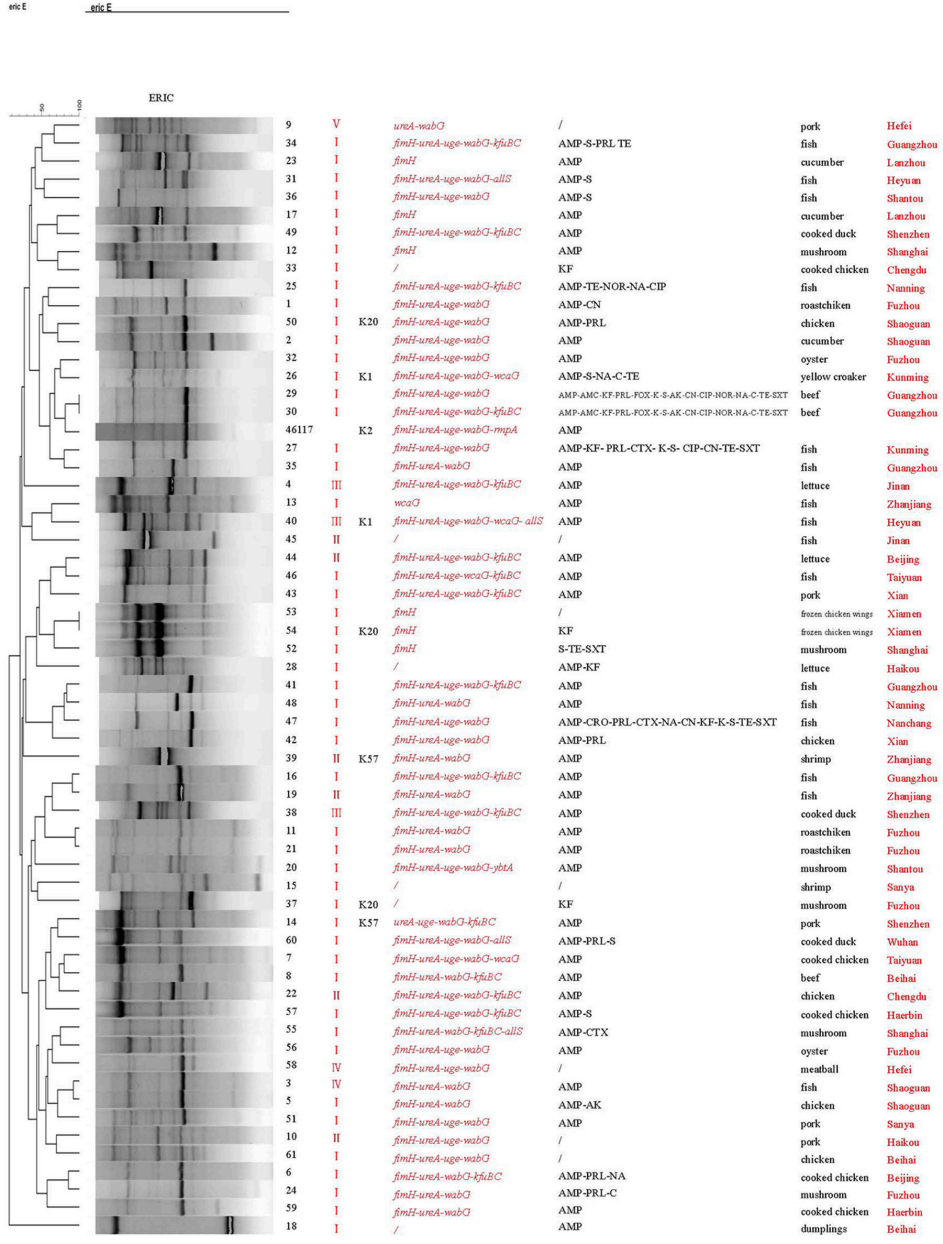
ERIC-PCR molecular fingerprint profiles of *Klebsiella pneumoniae*.

**Figure 3:**
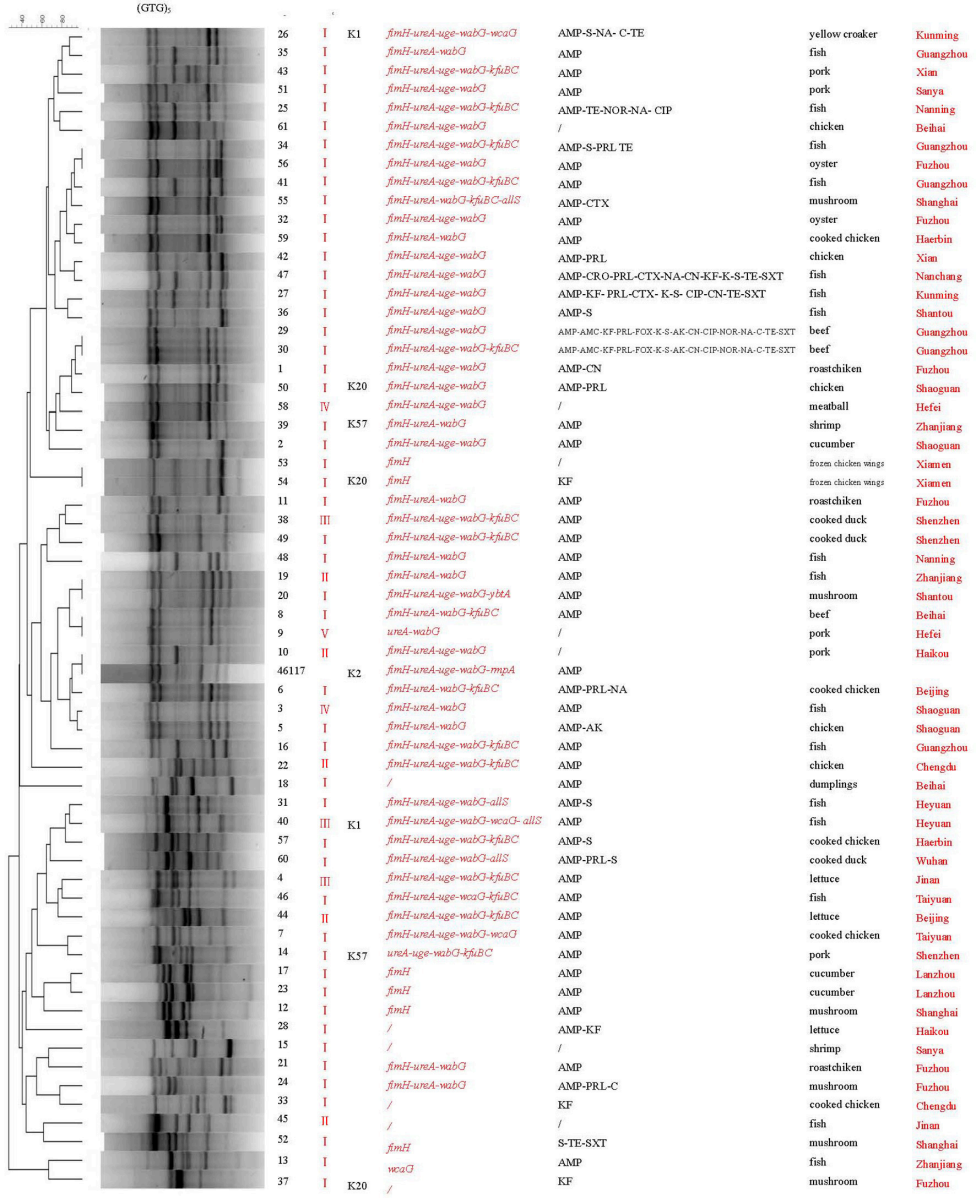
(GTG)_5_-PCR molecular fingerprint profiles of *Klebsiella pneumoniae*. AMP, Antibiotics: ampicillin; AMC, amoxycillin-clavulanicAcid; CAZ, ceftazidime; CTX, cefotaxime; CRO, ceftriaxone; SCF, cefoperazone/sulbactam; KF, cephalothin; PRL, piperacillin; TZP, piperacillin/tazobactam; IPM, imipenem; FOX, cefoxitin; CIP, ciprofloxacin; NA, nalidixic acid; NOR, norfloxacin; AK, amikacin; CN, gentamicin; K, kanamycin; S, streptomycin; C, chloramphenicol; SXT, trimethoprim-sulfamethoxazole; TE, tetracycline; /, susceptible Biotypes: I“7215773”; II “1215773”; III “7215773”; IV “ 5215373”; V “5214773”.

ERIC-PCR classified the 62 strains into 60 different patterns (Figure 2). Two isolates (numbers 53 and 54) obtained from frozen chicken wings in Xiamen exhibited identical patterns and carried the same virulence gene, *fimH*. Likewise, two isolates (numbers 29 and 30) obtained from a beef sample in Guangzhou also showed identical genetic profiles. All other isolates belonged to distinct genetic types. Two isolates (numbers 11 and 21) obtained from Fuzhou but different food samples exhibited similar genetic profiles and virulent gene profiles. Similar findings were observed for two isolates (numbers 3 and 5) obtained from Shaoguan and the other two isolates (numbers 50 and 2) obtained from Shaoguan.

(GTG)_5_-PCR analysis revealed that the food isolates and reference strain could be divided into 56 different genetic patterns (Figure 3). Again isolates 53 and 54 exhibited the same genetic profile, as did isolates 29 and 30. Some isolates with similar virulence genes but from different cities (isolates 34 and 56, 19 and 20, and 8 and 9) also yielded the same genetic profiles. One isolate (10) yielded a genetic pattern identical to that of the reference strain. With the exception of these six pairs of isolates with identical patterns, all other isolates showed unique genetic patterns. Some isolates obtained from the same cities (isolates 3 and 5, 17 and 23, 31 and 40) showed similar genetic profiles and virulent gene profiles, respectively.

## Discussion

### Biotyping, serotypes, and virulence gene distributions

Biotyping of *K. pneumoniae* is an important method of bacterial characterization for supplementing epidemiological studies. Our results showed that the biochemical profiles of the food isolates were diverse. We identified five different biotypes among the 62 isolates, among which “5215773” was the most prevalent biotype profile. However, no obvious correlations were observed between the biotype and other phenotypic factors, such as serotype and virulence factors. Therefore, the biotyping method should be combined with other methods to accurately study this pathogen.

Capsules are important virulence factors that are associated with the severity of infection. In our study, four capsular types (K1, K2, K20, and K54) were identified among the isolates. Most of the isolates were non-K1/K2 serotypes. This finding is inconsistent with those of previous studies. Wei et al. (2016) reported that capsule serotypes K1, K2, and K57 accounted for 82.1% of clinical high-virulence *K. pneumoniae* strains. In other studies, serotypes K1, K2, and K54 were found to be the most common capsular types among *K. pneumoniae* clinical isolates (Yu et al., 2008; Cheng et al., 2015). The difference in results may be related to the origins and geographic distributions of the isolates examined. Although K1/K2 are considered to be the most virulent serotypes, increasing clinical and epidemiological evidence suggests that some non-K1/K2 serotypes are also virulent and can cause various human infections (Yao et al., 2015; Yu et al., 2015). Therefore, the presence of these serotypes in foods still poses a potential public health risk. In addition, the strains with high virulent capsular types were mainly isolated from meat and aquatic products, indicating the high risk of these food types.

The results of the assessment of virulence factors suggested that the *fimH, ureA, uge*, and *wabG* genes were commonly distributed among food isolates, which is consistent with the results reported for clinical isolates (Yu et al., 2008; Calhau et al., 2014; Cheng et al., 2015). The presence of these genes in food isolates suggests the pathogenic potential of these isolates and a potential risk to human health. The *rmpA* gene is a putative virulence factor and has been found to be associated with highly virulent *K. pneumoniae* (Yu et al., 2006). Yu et al. (2007) found that *rmpA* is mainly prevalent among *K. pneumoniae* isolates categorized as capsular serotypes K1 and K2, causing internal infections with abscessation in Southeast Asia. Other studies have also reported that capsular serotypes K1 and K2 are often associated with the *rmpA* gene (Turton, 2010; Cheng et al., 2015). However, we did not detect the *rmpA* gene in serotype K1 strains. In addition, Yu et al. (2008) reported that there was a strong association between the *kfuB* and *allS* genes and K1 serotype isolates, with all K1 clinical isolates testing positive for *kfuB* and *allS*. However, in the present study, we found no obvious correlation between capsular serotype and any virulence genes. *kfuB* was not detected in K1 serotype isolates. Furthermore, *kfuB* and *allS* were present in isolates belonging to other serotypes. Our results indicate that the virulence profiles of the isolates were diverse and that virulence genes were commonly present in different capsular types. These findings may be related to the limited number of isolates in our study. In future studies, a greater number of isolates should be analyzed to assess the relationship between capsular type and virulence factors.

### Antibiotic resistance of *K. Pneumoniae*

The extensive use of antimicrobials has led to a high incidence of resistance in *K. pneumoniae* (Cao et al., 2014; Kim et al., 2015). The food isolates in our study showed a high prevalence of resistance to ampicillin. Resistance to piperacillin, cephalothin, streptomycin, and tetracycline was also common. These results are consistent with previous findings (Haryani et al., 2007; Hassan et al., 2011; Nawaz et al., 2012). Nawaz et al. (2012) reported that 47% of *K. pneumoniae* isolated from shrimp were resistant to trimethoprim/sulfamethaxazole and chloramphenicol. In our study, most isolates were susceptible to these two antibiotics. Quinolones are broad-spectrum antimicrobial agents that have been widely used in clinical medicine and in raising food-producing animals (such as chicken in China). Wu et al. (2016) found that 80.0% of isolates from chicken broilers were resistant to ciprofloxacin. Kim et al. (2015) also found that 26.3% of *K. pneumoniae* isolates from ready-to-eat vegetables were resistant to ciprofloxacin. In contrast, more than 90% of our isolates were susceptible to quinolone antibiotics, including ciprofloxacin, nalidixic acid, and norfloxacin. The third-generation cephalosporins, such as cefotaxime, ceftriaxone, and piperacillin, play an important role in clinical therapeutic use. In this study, most food isolates were susceptible to these antibiotics, indicating that third-generation cephalosporin antibiotics could be effective against food isolates. However, three isolates were found to be resistant to cefotaxime, which merits concern. These strains usually produce extended spectrum β-lactamases and often confer resistance to almost all β-lactam antibiotics, including 3rd and 4th generation cephalosporins and other kinds of antibiotics (quinolones, trimethoprim/sulfamethoxazole, and aminoglycosides). The presence of such strains in food may represent a significant threat to consumers, as these pathogens have been recorded to cause obstinate infections with increased morbidity and mortality (Warjri et al., 2015; Koovapra et al., 2016).

In addition, we also detected 11 multidrug-resistant (≥3 drugs) isolates. Multidrug-resistant strains are of great public health concern, as they may further complicate the treatment of human infections caused by *K. pneumoniae*. Multidrug resistance increases the risk of antimicrobial treatment failure in humans. Continuous surveillance of the antibiotic resistance of *K. pneumoniae* in foods is therefore needed.

### ERIC-PCR and (GTG)_5_-PCR molecular typing

Both ERIC-PCR and (GTG)_5_-PCR molecular typing results revealed a high degree of genetic diversity among the *K. pneumoniae* isolates from retail foods. Interestingly, a good concordance was observed between ERIC and (GTG)_5_ typing in four isolates. Isolates 53 and 54 from the same sample were 100% identical according to the two genetic typing methods. Likewise, isolates 29 and 30 from the same sample also showed identical genotyping patterns with both methods. These results reflect the reliability and accuracy of these molecular methods.

Further analysis of the associations between phenotypic types and molecular subtypes indicated that genotyping results generally correlated with virulence gene profiles. In (GTG)_5_-PCR typing, most isolates with similar or identical virulence gene profiles exhibited close genetic relationships (Figure 3). Similarly, in ERIC typing, some isolates (53, 54, and 52; 44, 46, and 43; 11 and 21; 15 and 37; 3 and 5; 10 and 61) carrying similar virulence factors were genetically related.

Based on the isolation sources, we found that some isolates obtained from the same geographic area showed similar genetic profiles and had a closer relationship. Additionally, some isolates from the same city were divided into different clusters in ERIC or (GTG)_5_ typing, indicating the genetic heterogeneity of these isolates. However, no clear correlation was observed between the genotyping patterns of isolates and food types, consistent with that of a previous study (Barus et al., 2013). The isolates with the same biotypes also exhibited diversity genetic profiles and were distributed in different clusters in ERIC or (GTG)_5_ typing. Some isolates with different biotypes were clustered together in ERIC and (GTG)_5_ profiles. Further studies are needed to confirm the relationship between biotypes and genotypes of *K. pneumoniae* with a larger number of food strains.

Compared with (GTG)_5_-PCR, ERIC-PCR exhibited a higher discriminatory ability to distinguish these isolates, as ERIC-PCR was able to differentiate even the isolates with the same (GTG)_5_ genetic profiles, including isolates 34 and 56, 19 and 20, and 8 and 9, which originated from different sources. Merging the results from the two fingerprinting methods enhanced detection of polymorphisms. Our analyses suggest that a combination of ERIC-PCR and (GTG)_5_-PCR is more effective for analysing the epidemiological and virulence characteristics of *K. pneumoniae*.

## Conclusions

Our results indicate that food-borne *K. pneumoniae* exhibit diverse virulence gene profiles, antibiotic resistance profiles, and genotypes. Highly virulent serotypes and multidrug-resistant isolates were present in foods. The potential health risks posed by such isolates should not be underestimated. Our findings highlight the need for increasing the surveillance of *K. pneumoniae* in foods. These data improve our understanding of the epidemiological and public health implications of this pathogen.

## Author contributions

SZ, GY, and QW: conceived and designed the experiments. SZ and GY: performed the experiments. SZ, GY, QY, and YH: analyzed the data. SZ, GY, QW, and JZ: contributed reagents, materials and analysis tools.

### Conflict of interest statement

The authors declare that the research was conducted in the absence of any commercial or financial relationships that could be construed as a potential conflict of interest.
