# Supplementary material for: Phenotypic and Genotypic Characterization of Klebsiella pneumoniae Isolated From Retail Foods in China
Source: Front Microbiol. 2018 Mar 1;9:289. doi: 10.3389/fmicb.2018.00289 (PMC5839057; doi:10.3389/fmicb.2018.00289)
Supplement: Supplementary file 1 [file Table1.docx]

Table S1 The distribution of serotypes, virulence genes, and biotypes of *Klebsiella pneumoniae* strains

|  | Strains | Serotypes | API 20 E profiles | Virulence genes | Food samples | Cities |
| --- | --- | --- | --- | --- | --- | --- |
| 1 | FZ36A2 |  | 5215773 | *fimH-ureA-uge-wabG* | roast chiken | Fuzhou |
| 2 | SG41B1 |  | 5215773 | *fimH-ureA-uge-wabG* | cucumber | Shaoguan |
| 3 | SG31C2 |  | 5215373 | *fimH-ureA-wabG* | fish | Shaoguan |
| 4 | JN31 |  | 7215773 | *fimH-ureA-uge-wabG-kfuBC* | lettuce | Jinan |
| 5 | SG23 |  | 5215773 | *fimH-ureA-wabG* | chicken | Shaoguan |
| 6 | BJ11 |  | 5215773 | *fimH-ureA-wabG-kfuBC* | cooked chicken | Beijing |
| 7 | TY38B3 |  | 5215773 | *fimH-ureA-uge-wabG-wcaG* | cooked chicken | Taiyuan |
| 8 | BH21A1 |  | 5215773 | *fimH-ureA-wabG-kfuBC* | beef | Beihai |
| 9 | HF45C1 |  | 5214773 | *ureA-wabG* | pork | Hefei |
| 10 | HK29 |  | 1215773 | *fimH-ureA-uge-wabG* | pork | Haikou |
| 11 | FZ27A3 |  | 5215773 | *fimH-ureA-wabG* | roast chiken | Fuzhou |
| 12 | SH19A1 |  | 5215773 | *fimH* | mushroom | Shanghai |
| 13 | ZJ07B2 |  | 5215773 | *wcaG* | fish | Zhanjiang |
| 14 | SZ01 | K57 | 5215773 | *ureA-uge-wabG- kfuBC* | pork | Shenzhen |
| 15 | SY27C1 |  | 5215773 | */* | shrimp | Sanya |
| 16 | GZYX06A1 |  | 5215773 | *fimH-ureA-uge-wabG-kfuBC* | fish | Guangzhou |
| 17 | LZ46A1 |  | 5215773 | *fimH* | cucumber | Lanzhou |
| 18 | BH17A3 |  | 5215773 | */* | dumplings | Beihai |
| 19 | ZJ30 |  | 1215773 | *fimH-ureA-wabG* | fish | Zhanjiang |
| 20 | ST46C1 |  | 5215773 | *fimH-ureA-uge-wabG- ybtA* | mushroom | Shantou |
| 21 | FZ27B3 |  | 5215773 | *fimH-ureA-wabG* | roast chiken | Fuzhou |
| 22 | CD23B2 |  | 1215773 | *fimH-ureA-uge-wabG- kfuBC* | chicken | Chengdu |
| 23 | LZ14A2 |  | 5215773 | *fimH* | cucumber | Lanzhou |
| 24 | FZ49A3 |  | 5215773 | *fimH-ureA-wabG* | mushroom | Fuzhou |
| 25 | NN28A1 |  | 5215773 | *fimH-ureA-uge-wabG- kfuBC* | fish | Nanning |
| 26 | KM14C1 | K1 | 5215773 | *fimH-ureA-uge-wabG-wcaG* | yellow croaker | Kunming |
| 27 | KM05 |  | 5215773 | *fimH-ureA-uge-wabG* | fish | Kunming |
| 28 | HK41 |  | 5215773 | */* | lettuce | Haikou |
| 29 | GZYX02A1 |  | 5215773 | *fimH-ureA-uge-wabG* | beef | Guangzhou |
| 30 | GZYX02A2 |  | 5215773 | *fimH-ureA-uge-wabG- kfuBC* | beef | Guangzhou |
| 31 | HY06 |  | 5215773 | *fimH-ureA-uge-wabG- allS* | fish | Heyuan |
| 32 | FZ23A2 |  | 5215773 | *fimH-ureA-uge-wabG* | oyster | Fuzhou |
| 33 | CD36 |  | 5215773 | */* | cooked chicken | Chengdu |
| 34 | GZYX28A1 |  | 5215773 | *fimH-ureA-uge-wabG- kfuBC* | fish | Guangzhou |
| 35 | GZYX29 |  | 5215773 | *fimH-ureA-wabG* | fish | Guangzhou |
| 36 | ST04B3 |  | 5215773 | *fimH-ureA-uge-wabG* | fish | Shantou |
| 37 | FZ34B2 | K20 | 5215773 | */* | mushroom | Fuzhou |
| 38 | SZ37C3 |  | 7215773 | *fimH-ureA-uge-wabG- kfuBC* | cooked duck | Shenzhen |
| 39 | ZJ04 | K57 | 1215773 | *fimH-ureA-wabG* | shrimp | Zhanjiang |
| 40 | HY29C1 | K1 | 7215773 | *fimH-ureA-uge-wabG-wcaG-allS* | fish | Heyuan |
| 41 | GZYX27 |  | 5215773 | *fimH-ureA-uge-wabG- kfuBC* | fish | Guangzhou |
| 42 | XA23A1 |  | 5215773 | *fimH-ureA-uge-wabG* | chicken | Xian |
| 43 | XA01 |  | 5215773 | *fimH-ureA-uge-wabG- kfuBC* | pork | Xian |
| 44 | BJ24B1 |  | 1215773 | *fimH-ureA-uge-wabG- kfuBC* | lettuce | Beijing |
| 45 | JN41 |  | 1215773 | */* | fish | Jinan |
| 46 | TY05 |  | 5215773 | *fimH-ureA-uge-wcaG- kfuBC* | fish | Taiyuan |
| 47 | NC28B1 |  | 5215773 | *fimH-ureA-uge-wabG* | fish | Nanchang |
| 48 | NN30 |  | 5215773 | *fimH-ureA-wabG* | fish | Nanning |
| 49 | SZ34 |  | 5215773 | *fimH-ureA-uge-wabG- kfuBC* | cooked duck | Shenzhen |
| 50 | SG23B1 | K20 | 5215773 | *fimH-ureA-uge-wabG* | chicken | Shaoguan |
| 51 | SY35A1 |  | 5215773 | *fimH-ureA-uge-wabG* | pork | Sanya |
| 52 | SH48A1 |  | 5215773 | *fimH* | mushroom | Shanghai |
| 53 | XM12A2 |  | 5215773 | *fimH* | frozen chicken wings | Xiamen |
| 54 | XM12A1 | K20 | 5215773 | *fimH* | frozen chicken wings | Xiamen |
| 55 | SH19A2 |  | 5215773 | *kfuBC-fimH-ureA-wabG- allS* | mushroom | Shanghai |
| 56 | FZ06A2 |  | 5215773 | *fimH-ureA-uge-wabG* | oyster | Fuzhou |
| 57 | HRB39A1 |  | 5215773 | *fimH-ureA-uge-wabG- kfuBC* | cooked chicken | Haerbim |
| 58 | HF09C1 |  | 5215373 | *fimH-ureA-uge-wabG* | meatball | Hefei |
| 59 | HEB38 |  | 5215773 | *fimH-ureA-wabG* | cooked chicken | Haerbim |
| 60 | WH11 |  | 5215773 | *fimH-ureA-uge-wabG- allS* | cooked duck | Wuhan |
| 61 | BH25A1 |  | 5215773 | *fimH-ureA-uge-wabG* | chicken | Beihai |
| 46117 |  | K2 | 5215773 | *fimH-ureA-uge-wabG- rmpA* |  |  |
